# Psychometric properties of the Nomophobia Questionnaire (NMP-Q) in Peruvian adolescents

**DOI:** 10.3389/fpsyg.2024.1399328

**Published:** 2025-01-29

**Authors:** Joel Figueroa-Quiñones, Miguel Ipanaqué-Zapata, Oriana Rivera-Lozada, Giusepi Zevallos Mayanga, Diana Gonzales Diaz

**Affiliations:** ^1^Universidad Autónoma de Ica, Ica, Peru; ^2^Universidad Señor de Sipán, Chiclayo, Peru

**Keywords:** adolescents, measurement invariance, psychometric properties, NMP-Q, nomophobia

## Abstract

**Background:**

Adolescence and technological devices have a close relationship during this stage in which anxiety about using a cell phone increases when it is not available. The Nomophobia Scale (NMP-Q) is a measure that evaluates anxiety behaviors when being without a mobile phone.

**Objective:**

To evaluate the psychometric properties of the Nomophobia Questionnaire (NMP-Q) in Peruvian adolescents.

**Methods:**

An instrumental study was conducted and 900 adolescents of both sexes, between 12 and 17 years old, living in northern, rural, and eastern regions of Peru were evaluated. A confirmatory factor analysis was carried out to determine the structure and the structural invariance of the measures according to age was evaluated and the reliability was estimated by means of the Omega reliability coefficient.

**Results:**

The four--factor structure composed of 20 items was confirmed with optimal goodness-of-fit indices (CFI = 0.992; TLI = 0.991; SRMR = 0.053; RMSEA = 0.039). The MIMIC models reported invariance for age groups (ΔCFI <0.01, ΔRMSEA <0.015). The omega reliability coefficients ranged between 0.84 and 0.90.”

**Conclusion:**

The Peruvian version of the NMP-Q (20 items) has shown adequate psychometric properties to assess nomophobia in the adolescent population.

## 1 Introduction

The massive use of the Internet in the population has become a hallmark of modern society (Khiabany, 2010). The main advantage of using the Internet is the possibility of connecting people from all corners of the world, breaking the barriers of distance and geography (Marcoccia, 2012). In addition, services related to education, health, work or entertainment are offered through the Internet (Mihalca et al., 2021; Okyere, 2022). Likewise, the possibility of connecting to the Internet from any device such as a smartphone, laptop, or tablet facilitates the access and use of these technologies (Villanti et al., 2017).

It is estimated that worldwide there are more than 7 billion people who have a smartphone (Statista, 2023a), with China and India being the countries with the highest proportion of mobile users (Statista, 2022). At the Latin American level, Brazil and Mexico are the countries with the highest number of users (Statista, 2023b). In fact, a study has reported that users check their mobile phones an average of 84 times per day and spend approximately 5 h daily using their devices (Andrews et al., 2015).

The literature suggests that excessive use of mobile phones by users has developed an irrational fear, called nomophobia, when access to the mobile phone is not available for much of the day (Pérez Cabrejos et al., 2021). People with nomophobia show obsessive-type behavior such as constantly checking their cell phone and excessive concern about their internet connection and cell phone battery (Yildirim et al., 2016). A systematic review reported that between 6 and 73% of mobile phone users suffer from nomophobia (León-Mejía et al., 2021). Most studies have found that this phenomenon is more frequent in the young population, given that they acquire a cell phone from an early age (Gurbuz and Ozkan, 2020; Rémen and Lacour, 2018; Terras and Ramsay, 2016; Limone and Toto, 2022).

There are several studies that reveal the problem of nomophobia, and its association with the presence of mental health problems in the adolescent population (Sharma et al., 2019). For example, a report with Croatian students revealed that the majority were afraid of losing or being without their mobile phone, expressing anxiety, anguish, nervousness and discomfort given the need for a mobile connection (Santl et al., 2022). Another study in Turkey identified that anxiety, phobia, and depression were correlated with adolescent nomophobia (Kuscu et al., 2021). And a study in Brazil revealed that participants with nomophobia reported worse mental health scores and academic performance (Kubrusly et al., 2021).

To evaluate nomophobia, various instruments have been developed and validated. For example, the mobile phone participation questionnaire (MPIQ) developed in Spain for the university population (Argumosa-Villar et al., 2017). In Turkey, the Fırat Single Factor Nomophobia Scale was conducted to assess nomophobia in the adult population (Kanbay et al., 2022) or the self-rated version of the Nomophobia Severity Index (NSI-SR) (Mathew and Bivin, 2012). However, the most used scale is the Nomophobia Questionnaire (NMP-Q), which has been widely applied in research around the world (Yildirim and Correia, 2015).

The NMP-Q assesses nomophobia, an anxiety disorder characterized by an intense, irrational and disproportionate fear of not being able to use a mobile phone (Yildirim and Correia, 2015). The NMP-Q evaluates four dimensions: (a) Concern about lack of access to information: which involves the fear of losing access to information through the mobile phone. (b) Concern about the lack of connection with others, which measures the fear of losing contact with family, friends, and colleagues; (c) Concern about the loss of digital identity which evaluates the fear of losing access to social networks and other online services and (d) Concern about giving up convenience, which investigates the fear of being unable to comfortably manage the use of the mobile phone (Yildirim and Correia, 2015).

The NMP-Q is composed of 20 clear and understandable items evaluated with a 7-point Likert scale and scores ranging between 20 and 140, with higher scores corresponding to greater intensity of nomophobia. This instrument has also been adapted and validated in other populations. In Portugal, the validation was conducted in an adult population, reporting reliability through the global alpha of 0.96, and its factors showed factor loadings greater than 0.87 (Galhardo et al., 2020). Another study in China, conducted with university students, found factor loadings in its four dimensions exceeding 0.81 (Gao et al., 2020). In Germany (Coenen and Görlich, 2022) and Spain (Roque Hernández and Guerra Moya, 2022), adaptations of the NMP-Q were made, and evaluations of the psychometric (RRID:SCR_024295) properties of its optimal structure reported the formation of four factors with reliability above 0.70. Furthermore, studies have been conducted evaluating the psychometric properties of confirmatory factor analysis and measurement invariance of the questionnaire, reporting adequate values. A study conducted across 11 countries presented results of measurement invariance of the NMP-Q within age and gender groups (Zhang et al., 2024). Another study conducted in four Asian countries presented results where fit indices were optimal (TLI, CFI > 0.90, SRMR <0.03, and RMSEA <0.08), and measurement invariance by gender was confirmed (Li et al., 2023).

In Peru, 89.3% of the population over 6 years of age uses a cell phone and the age group with the greatest accessibility to cell phones and the Internet is between 12 and 24 years of age (Instituto Nacional Estadística e Informática, 2020). A study conducted with Peruvian medical students revealed that 25.7% presented moderate nomophobia associated with symptoms of anxiety or depression (Copaja-Corzo et al., 2022). Another study in the Peruvian capital confirmed the four-dimensional structure of the NMP-Q, and a reliability with a Cronbach’s Alpha coefficient of 0.964, indicating that the scores guarantee the internal consistency of the instrument (Franco-Guanilo et al., 2022). However, this study was validated in a university population, so its application in other groups could be counterproductive. In fact, other study applied with Peruvian adolescents have evaluated the level of nomophobia using the NMP-Q with a partial report of its validity through expert judgment, assuming the guarantee of its reliability and validity of measurement (Manchego, 2022), however the use of instruments in this way produces inconsistent and unreliable measurements, distorting the real result of the variable being evaluated (Carretero-Dios and Pérez, 2007), It is also important to mention that within the existing literature, there is no evaluation of measurement invariance, which represents a clear weakness and gap for implementing the instrument in an objective and reliable manner.

As stated, the aim of this study was to evaluate the psychometric properties of the NMP-Q in Peruvian adolescents. Additionally, it had the following specific objectives: (a) to confirm the structure of the NMP-Q, (b) to determine the reliability of the scale, and (c) to explore measurement invariance across subgroups based on key sociodemographic indicators (sex and region), as these variables may affect validity due to differences in attitudes, behaviors, and/or cultural factors.

## 2 Methods

### 2.1 Design study

A cross-sectional and psychometric study was conducted to evaluate the psychometric properties of the Nomophobia Questionnaire (NMP-Q) in Peruvian adolescents (Sánchez and Echeverry, 2004).

### 2.2 Participants

The participants consisted of 900 secondary school adolescents from the coast (Chiclayo, *n* = 300), highlands (Cajamarca, *n* = 300) and jungle (Pucallpa, *n* = 300) of public educational institutions in Peru. Participants were selected by non-probabilistic purposive sampling, and included those who: (1) were between 12 and 17 years old, (2) agreed to participate in the study and their parents gave informed consent. There were no missing data among the respondents; that is, the entire sample was included in the data analysis. The sample size followed the recommendations proposed for instrument validation, 20 participants per item (31). To ensure sufficient statistical power and variability in the data, we sought to double the suggested number, while acknowledging that the non-probabilistic sampling limits the generalisability of the findings. The average age of the participants was 14.3 years and the majority of participants were female (50.4%).

### 2.3 Variables and instruments

The Nomophobia Questionnaire (NMP-Q) was originally developed by Yildirim and Correia (2015) in the USA. This instrument has 20 items and four factors. The response form is a 7-point Likert-type scale, from 1 (strongly disagree) to 7 (strongly agree). Scores range from a minimum of 20 to a maximum of 140 points. The overall reliability of the NMP-Q in Peruvian population is *α* = 0.96 and of the four dimensions 0.94, 0.93, 0.86 and 0.87, respectively (Franco-Guanilo et al., 2022).

The sociodemographic variables included in the study were age (years), place of residence (coast, highlands and jungle), sex (male or female).

### 2.4 Procedures

The procedures and international guidelines suggested by international organizations for the adaptation and validation of measurement instruments were applied (American Educational Research Association, American Psychological Association, and National Council on Measurement in Education, 2014).

The Nomophobia Questionnaire (NMP-Q) was translated from English into Spanish by a bilingual expert and subsequently back-translated into English to verify its equivalence. Finally, a preliminary version was produced after reaching a consensus among the translators. Additionally, professional experts were consulted to ensure the coherence, clarity, and relevance of the items in the English version (González-Cabrera et al., 2017). Subsequently, the instrument was applied in the Peruvian context in a specific city, demonstrating good reliability and psychometric validity indicators; however, this was limited to an exploratory factor analysis (Franco-Guanilo et al., 2022). In this research project, the content was validated using a qualitative approach. Specialists were engaged to confirm that the instrument, adapted to Peruvian Spanish and designed for adolescents, was appropriate for the study population in this investigation.

Authorization was requested from the principals of the three cities and the parents of the adolescents were informed of the objective of the study through an informed consent form. The final version of the Nomophobia Questionnaire (NMP-Q) was shared in the educational classrooms in person and collectively during class time, by trained evaluators in each institution in each city, who remained during the evaluation to answer the adolescents’ questions. The questionnaire included the informed consent of the adolescents, the objectives of the study and the scales. The application was carried out during May and June 2023 and the answers were digitalized and saved in Excel files protected with passwords and accessible only to the principal investigator.

### 2.5 Analysis statistical

Quantitative sociodemographic variables were analyzed with central tendency statistics to report the mean and standard deviation, while categorical variables were presented as frequencies and percentages.

The four-factor structure of the NMP-Q was reviewed through a confirmatory factor analysis, using the Diagonally Weighted Least Squares (DWLS) estimator suggested for categorical and ordinal variables (Brown, 2014). The comparative fit index (CFI > 0.90), the Tucker-Lewis index (TLI > 0.90), the standardized root mean square residual (SRMR<0.06), the root mean square error of approximation (RMSEA<0.08) were reviewed with their respective values considered as adequate (Keith, 2014; Schumacker and Lomax, 2004).

Reliability was evaluated by means of internal consistency through the omega coefficient, given that it is recommended when the tau equivalence model is not fulfilled. Values above 0.7 were considered optimal to show reliability (Dunn et al., 2014).

Measurement invariance was assessed to determine whether comparisons between the groups of the covariates (sex and region) were feasible, using a series of nested models (configural, metric, scalar, and residual). To evaluate the four models, the variation in goodness-of-fit indicators (CFI and RMSEA) between the restricted models and the previous less restricted model was considered, indicating that the added restrictions did not significantly affect the model. Invariance was considered to exist when the variation in CFI was less than or equal to 0.010 and in RMSEA less than or equal to 0.015 (Chen, 2007; Arrindell et al., 2022).

### 2.6 Ethics

This study was reviewed by the Research Ethics Committee of the Universidad Señor of Sipan. Likewise, the ethical principles for research with human beings proposed in the Declaration of Helsinki were applied, through respect for the participants, justice and beneficence through the application of informed consent and informed assent to the parents and participants, respectively, (General Assembly of the World Medical Association, 1964). Likewise, the data were confidential and protected in password-protected files.

## 3 Results

### 3.1 Descriptive analysis of the items

Table 1 shows the analysis of the 20 items proposed in the NMP-Q scale. It is observed that item 11 has the highest mean score and item 8 the maximum variability (*M* = 4.02; SD =2.04). Likewise, with respect to the skewness and kurtosis scores of the items, values lower than ±2 are observed and the correlation of the items with the rest fluctuates between 0.51 and 0.73.

**Table 1:** Items analysis for the nomophobia scale (NMP-Q).

	M	SD	Sk	Ku	Item-rest correlation
Item 1	3.01	1.80	0.51938	−0.921	0.651
Item 2	3.46	1.89	0.28091	−1.258	0.581
Item 3	3.05	1.86	0.65446	−0.796	0.577
Item 4	3.34	1.81	0.26861	−1.143	0.685
Item 5	2.79	1.77	0.80255	−0.499	0.579
Item 6	2.69	1.68	0.90157	−0.170	0.626
Item 7	3.35	1.93	0.3443	−1.183	0.713
Item 8	3.38	2.04	0.36787	−1.253	0.641
Item 9	3.51	2.00	0.23797	−1.323	0.706
Item 10	3.80	1.93	0.00218	−1.324	0.683
Item 11	4.02	2.01	−0.13101	−1.401	0.645
Item 12	3.20	1.87	0.49768	−0.968	0.735
Item 13	3.73	1.93	0.07345	−1.276	0.734
Item 14	3.42	1.85	0.28569	−1.138	0.725
Item 15	3.70	1.93	0.19416	−1.183	0.609
Item 16	2.85	1.77	0.70446	−0.605	0.616
Item 17	2.80	1.78	0.77649	−0.486	0.617
Item 18	2.86	1.77	0.79620	−0.437	0.650
Item 19	2.59	1.66	0.91919	−0.184	0.515
Item 20	2.98	1.80	0.58429	−0.805	0.651

M, media; SD, Standard deviation; Sk, skewness; Ku, Kurtosis.

### 3.2 Confirmatory factor analysis

**Figure 1:**
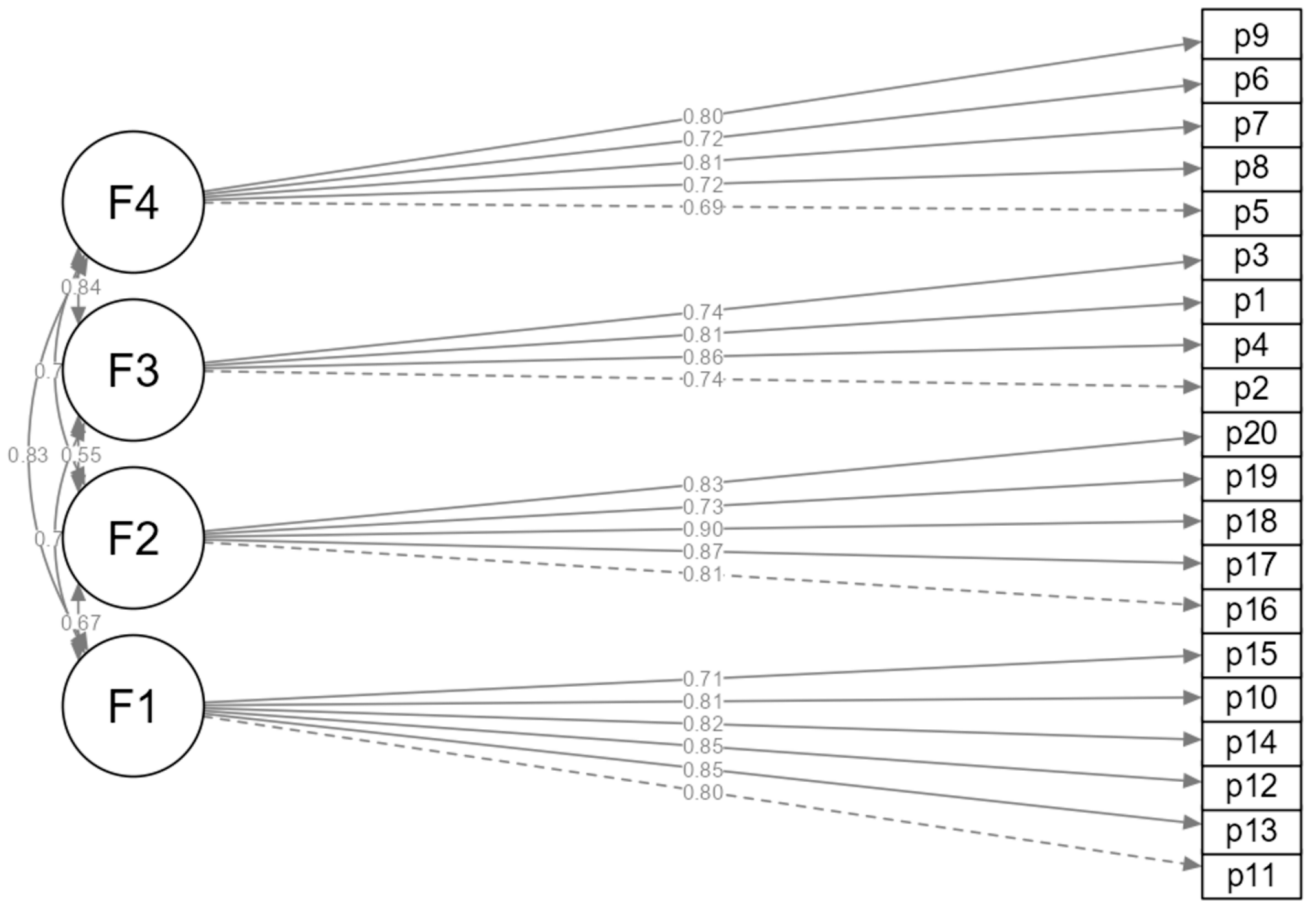
Factor structure of the NMP-Q.

The NMP-Q shows a 04-factor structure with optimal goodness-of-fit indices (CFI = 0.992; TLI = 0.991; SRMR = 0.053; RMSEA = 0.039) (Table 2). Likewise, the model loaded a minimum of *λ* = 0.69 and a maximum of *λ* = 0.90 to the NMP-Q items (Figure 1).

**Table 2:** Goodness of fit of the NMP-Q measurement model.

Model	Goodness of fit index	General (*N* = 900)
04 dimensions	χ2	1041
	CFI	0.992
	TLI	0.991
	SRMR	0.053
	RMSEA	0.077

*X*^2^(df) for model versus baseline. CFI, Comparative fit index; TLI, Tucker-Lewis index; SRMR, Standardized root mean squared residual; RMSEA, Root mean squared error of approximation.

### 3.3 Reliability

The NMP-Q reported an optimal reliability, with Omega values of 0.90 and intervals between 0.88 and 0.891 in its factor 1. Likewise, in its factor 2 it reported a *ω* = 0.89 (0.88–0.90); in factor 3 it revealed a *ω* = 0.84 (0.83–0.86) and factor 4 found a *ω* = 0.84 (0.82–0.85).

### 3.4 Measurement invariance

The invariance analysis was performed according to covariates (sex and region) to determine whether the groups were similar and comparable. Both males and females achieved invariance in the three models (metric, scalar, and residual), making them comparable to each other (ΔCFI ≤0.010 and ΔRMSEA ≤0.015). Likewise, the regions of the coast, jungle, and mountains reported measurement invariance, ensuring that the NMP-Q instrument was comparable among them (ΔCFI ≤0.010 and ΔRMSEA ≤0.015) (Table 3).

**Table 3:** Measurement invariance.

Model	CFI	RMSEA	ΔCFI	ΔRMSEA
Measurement according to sex				
Configural invariance	0.982	0.07	NA	NA
Metrict invariance	0.988	0.061	0.006	0.009
Scalar invariance	0.989	0.062	0.001	0.001
Residual invariance	0.99	0.063	0.001	0.001
Measurement according to city				
Configural invariance	0.966	0.107	NA	NA
Metrict invariance	0.968	0.096	0.002	0.011
Scalar invariance	0.959	0.097	0.009	0.001
Residual invariance	0.951	0.099	0.008	0.002

CFI, Comparative fit index; RMSEA, Root mean squared error of approximation; Δ = difference.

## 4 Discussion

The study sought to evaluate the psychometric properties of the NMP-Q in a sample of adolescents from different regions of Peru. The Peruvian version of the NMP-Q confirmed a four-component model that reports adequate goodness-of-fit indices and good reliability and invariance of measurement by age group.

The Peruvian version of the NMP-Q has psychometric properties similar to those of the original NMP-Q (Yildirim and Correia, 2015). In both validations, the internal structure of the NMP-Q maintained its four factors, that is, a four-structure model composed of 20 items to assess distress in the absence of control or contact with the cell phone. The conceptualization of all items is similar to that validated in other regions. For example, a study with youth from China, Iran, Bangladesh and Pakistan shows similar fit rates (TLI = 0.937; CFI = 0.945; SRMR = 0.040; RMSEA = 0.062) in the validation model (Li et al., 2023). Likewise, another study with the Mexican and Spanish population revealed an optimal internal structure of 04 factors (Caba-Machado et al., 2024).

In our study, the Peruvian version of the NMP-Q reported invariance by age group. This result is consistent with the results of the validation of the NMP-Q in Spanish adolescents CFI < 0.01 (León-Mejía et al., 2021). Similarly, the Mexican population has reported adequate invariance values in adolescents aged 14 to 19 years (Caba-Machado et al., 2024). These results indicate that the NMP-Q can be used to obtain equivalent and comparable results in Peruvian adolescents aged 12–17 years. Detecting invariance between age groups with the NMP-Q is important because invariant measures are necessary to better understand how these characteristics might influence the experience of distress in the absence of connection and control over their cell phones among adolescents.

The reliability of the NMP-Q in the sample of Peruvian adolescents was good. This result is consistent with a study of Portuguese adolescents, which reported an *α* = 0.96 in its 4-factor model (Galhardo et al., 2020). Likewise, the 20-item NMP-Q has shown optimal internal consistency in the Italian population with an alpha coefficient of 0.95 (Adawi et al., 2018). Although, in our study the reliability value was slightly lower, this could be due to the fact that previous studies the estimator used for internal consistency was Cronbach’s alpha which may have underestimated the reported value (Deng and Chan, 2017), whereas in our study the Omega index was used, which according to Flora (2020) reports more accurate estimates.

The Peruvian version of the NMP-Q provides solid psychometric evidence, suggesting that the instrument is suitable for use in educational contexts, specifically with adolescent students. Although this study did not evaluate the validity and reliability in clinical populations, the results indicate that the NMP-Q is a viable tool for assessing nomophobia in the school setting.

This is the first study to show psychometric evidence of the NMP-Q for nomophobia assessments in Peruvian adolescents. In addition, the study was conducted in a number of samples from representative regions of the country (Coast, Highlands and Jungle) and revealed age invariance. However, it is important to mention some limitations found in the study. First, although the sample of 900 people, representative of three regions of the country, was not randomly selected, which limits the generalization of the results at a national level, this geographically diverse approach allows for a more representative analysis within the three studied regions. Therefore, future studies with a broader scope should apply random sampling at a national level to expand the psychometric evidence. Additionally, it is necessary to validate the instrument in clinical populations within their respective contexts. Despite this, the NMP-Q has proven to be valid and reliable for assessing problems related to nomophobia. Finally, new studies are also needed to analyze the concurrent and discriminant validity of the NMP-Q, as well as its convergent, predictive, and known-groups validity, in addition to test–retest reliability, to evaluate how it relates to other similar measures.

## 5 Conclusion

In conclusion, the psychometric evidence found in the study supports the use of the NMP-Q in adolescents in Peru. The 20-item, four-factor structure of the NMP-Q was consistent. Likewise, reliability was optimal in all its factors and the invariance of the measurement suggested that people of different ages (12–17 years) interpreted the 20 items of the NMP-Q in a similar way. In this sense, the Peruvian version of the NMP-Q seems to be a solid instrument to assess nomophobia in this population.

## Data Availability

The raw data supporting the conclusions of this article will be made available by the authors, without undue reservation.
