# Supplementary material for: Psychometric properties of the Nomophobia Questionnaire (NMP-Q) in Peruvian adolescents
Source: Front Psychol. 2025 Jan 29;15:1399328. doi: 10.3389/fpsyg.2024.1399328 (PMC11813904; doi:10.3389/fpsyg.2024.1399328)
Supplement: Supplementary file 1 [file Data_Sheet_1.docx]

Supplementary Material

# Supplementary Data

## Supplementary Figures

| **Nomophobia Questionnaire (NMP-Q) Peruvian version in Spanish**  Please indicate the extent to which you agree or disagree with each question regarding your cell phone. | | | | | | | | |
| --- | --- | --- | --- | --- | --- | --- | --- | --- |
|  | | | | | | | | |
|  | **Totalmente en desacuerdo** | **En desacuerdo** | **Un poco en desacuerdo** | **Ni de acuerdo ni en desacuerdo** | **Un poco de acuerdo** | **De acuerdo** | **Totalmente de acuerdo** |  |
| 1. Me sentiría incómodo sin acceso constante a la información a través de mi teléfono celular |  |  |  |  |  |  |  |  |
| 2. Me molestaría no poder buscar información en mi teléfono celular cuando quisiera hacerlo |  |  |  |  |  |  |  |  |
| 3. Estaría nervioso si no pudiese obtener noticias (por ejemplo, eventos, clima, etc.) en mi teléfono celular |  |  |  |  |  |  |  |  |
| 4. Me molestaría no poder usar mi teléfono celular y/o sus capacidades cuando quisiera hacerlo |  |  |  |  |  |  |  |  |
| 5. Me asustaría quedarme sin batería en mi teléfono celular |  |  |  |  |  |  |  |  |
| 6. Entraría en pánico si me quedara sin saldo o llegara al límite mensual de uso de datos |  |  |  |  |  |  |  |  |
| 7. Si me quedara sin señal de datos o no pudiera conectarme a Wi-Fi, revisaría constantemente si tengo señal o pudiera conectarme a una red Wi-Fi |  |  |  |  |  |  |  |  |
| 8. Si no pudiera usar mi teléfono celular, tendría miedo de quedar perdido en algún lugar |  |  |  |  |  |  |  |  |
| 9. Si estuviera un tiempo sin revisar mi teléfono celular, tendría deseos de revisarlo |  |  |  |  |  |  |  |  |
| 10. Me sentiría ansioso si no pudiese comunicarme rápidamente con mi familia y/o amigos |  |  |  |  |  |  |  |  |
| 11. Estaría preocupado si mi familia y/o amigos no pudiesen comunicarse conmigo |  |  |  |  |  |  |  |  |
| 12. Estaría nervioso si no pudiera recibir mensajes de texto y llamadas |  |  |  |  |  |  |  |  |
| 13. Estaría ansioso si no pudiera mantenerme en contacto con mi familia y/o amigos |  |  |  |  |  |  |  |  |
| 14. Estaría nervioso si no supiera si alguien habría intentado comunicarse conmigo |  |  |  |  |  |  |  |  |
| 15. Estaría ansioso si mi conexión constante con mi familia y amigos se rompiera |  |  |  |  |  |  |  |  |
| 16. Estaría nervioso si estuviera desconectado de mi identidad en línea |  |  |  |  |  |  |  |  |
| 17. Me sentiría incómodo si no pudiese mantenerme al día con las redes sociales |  |  |  |  |  |  |  |  |
| 18. Me sentiría incómodo si no pudiese revisar mis notificaciones y redes en línea |  |  |  |  |  |  |  |  |
| 19. Me sentiría ansioso si no pudiese revisar mis mensajes de correo electrónico |  |  |  |  |  |  |  |  |
| 20. Me sentiría raro porque no sabría qué hacer sin conectividad |  |  |  |  |  |  |  |  |
| **Nomophobia Questionnaire (NMP-Q) Peruvian version in English**  Please indicate the extent to which you agree or disagree with each question regarding your cell phone. | | | | | | | | |
|  | | | | | | | | |
|  | **Strongly Disagree** | **Disagree** | **A little disagree** | **Neither agree nor disagree** | **A little agree** | **Agree** | **Strongly Agree** |  |
| 1. I would feel uncomfortable without constant access to information through my cell phone |  |  |  |  |  |  |  |  |
| 2. It would bother me if I couldn't look up information on my cell phone when I wanted to. |  |  |  |  |  |  |  |  |
| 3. I would be nervous if I couldn't get news (e.g. events, weather, etc.) on my cell phone |  |  |  |  |  |  |  |  |
| 4. I would be upset if I couldn't use my cell phone and/or its capabilities when I wanted to. |  |  |  |  |  |  |  |  |
| 5. I would be scared of running out of battery on my cell phone |  |  |  |  |  |  |  |  |
| 6. I would panic if I ran out of balance or reached my monthly data usage limit |  |  |  |  |  |  |  |  |
| 7. If I lost my data signal or couldn't connect to Wi-Fi, I would constantly check to see if I had a signal or could connect to a Wi-Fi network. |  |  |  |  |  |  |  |  |
| 8. If I couldn't use my cell phone, I would be afraid of getting lost somewhere. |  |  |  |  |  |  |  |  |
| 9. If I didn't check my cell phone for a while, I would want to check it. |  |  |  |  |  |  |  |  |
| 10. I would feel anxious if I couldn't communicate quickly with my family and/or friends |  |  |  |  |  |  |  |  |
| 11. I would be worried if my family and/or friends couldn't contact me |  |  |  |  |  |  |  |  |
| 12. I would be nervous if I couldn't receive texts and calls |  |  |  |  |  |  |  |  |
| 13. I would be anxious if I couldn't keep in touch with my family and/or friends |  |  |  |  |  |  |  |  |
| 14. I would be nervous if I didn't know if someone had tried to contact me |  |  |  |  |  |  |  |  |
| 15. I would be anxious if my constant connection with my family and friends broke down. |  |  |  |  |  |  |  |  |
| 16. I would be nervous if I were disconnected from my online identity. |  |  |  |  |  |  |  |  |
| 17. I would feel uncomfortable if I couldn't keep up with social media. |  |  |  |  |  |  |  |  |
| 18. I would feel uncomfortable if I couldn't check my notifications and networks online |  |  |  |  |  |  |  |  |
| 19. I would feel anxious if I couldn't check my emails. |  |  |  |  |  |  |  |  |
| 20. I would feel weird because I wouldn't know what to do without connectivity |  |  |  |  |  |  |  |  |
